# Clapp: Visiting List

**Published:** 1883-01

**Authors:** 


Otis Clapp & Son's Visiting List and Prescribing Record. Perpetual. Price, $2.00. Otis Clapp & Sons: Boston and Providence.

The neat "Visiting List" of this firm is well known. The size is convenient and the get-up elegant.

